# Breast cancer risk factors and a novel measure of volumetric breast density: cross-sectional study

**DOI:** 10.1038/sj.bjc.6604122

**Published:** 2007-12-18

**Authors:** M Jeffreys, R Warren, R Highnam, G Davey Smith

**Affiliations:** 1 Centre for Public Health Research, Massey University – Wellington Campus, Private Box 756, Wellington, New Zealand; 2 Department of Radiology, Level 5, Box 219, Addenbrooke's Hospital, Hills Road, Cambridge, UK; 3 Highnam Associates, Wellington, New Zealand; 4 Department of Social Medicine, University of Bristol, Canynge Hall, Whiteladies Road, Bristol, UK

**Keywords:** breast cancer, mammographic density, SMF, risk factors

## Abstract

We conducted a cross-sectional study nested within a prospective cohort of breast cancer risk factors and two novel measures of breast density volume among 590 women who had attended Glasgow University (1948–1968), replied to a postal questionnaire (2001) and attended breast screening in Scotland (1989–2002). Volumetric breast density was estimated using a fully automated computer programme applied to digitised film-screen mammograms, from medio-lateral oblique mammograms at the first-screening visit. This measured the proportion of the breast volume composed of dense (non-fatty) tissue (Standard Mammogram Form (SMF)%) and the absolute volume of this tissue (SMF volume, cm^3^). Median age at first screening was 54.1 years (range: 40.0–71.5), median SMF volume 70.25 cm^3^ (interquartile range: 51.0–103.0) and mean SMF% 26.3%, s.d.=8.0% (range: 12.7–58.8%). Age-adjusted logistic regression models showed a positive relationship between age at last menstrual period and SMF%, odds ratio (OR) per year later: 1.05 (95% confidence interval: 1.01–1.08, *P*=0.004). Number of pregnancies was inversely related to SMF volume, OR per extra pregnancy: 0.78 (0.70–0.86, *P*<0.001). There was a suggestion of a quadratic relationship between birthweight and SMF%, with lowest risks in women born under 2.5 and over 4 kg. Body mass index (BMI) at university (median age 19) and in 2001 (median age 62) were positively related to SMF volume, OR per extra kg m^−2^ 1.21 (1.15–1.28) and 1.17 (1.09–1.26), respectively, and inversely related to SMF%, OR per extra kg m^−2^ 0.83 (0.79–0.88) and 0.82 (0.76–0.88), respectively, *P*<0.001. Standard Mammogram Form% and absolute SMF volume are related to several, but not all, breast cancer risk factors. In particular, the positive relationship between BMI and SMF volume suggests that volume of dense breast tissue will be a useful marker in breast cancer studies.

The magnitude of the relationship between breast density and breast cancer has led to the use of breast density as a biomarker for breast cancer risk (Boyd *et al*, 1997, 1998a; Warren, 2004). Investigation of the relationship between risk factors and breast density can aid our understanding of aetiology. Many breast cancer risk factors are positively correlated with breast density, for example, birthweight (Cerhan *et al*, 2005), height (Gram *et al*, 1997; Boyd *et al*, 1998b), parity (Vachon *et al*, 2000) and age at first birth (El-Bastawissi *et al*, 2000). Users of hormone-replacement therapy have significantly higher levels of breast density (Sala *et al*, 2000; Vachon *et al*, 2000), whereas women on tamoxifen (Atkinson *et al*, 1999) have lower levels. There are two notable exceptions to the generalisation that risk factors also increase the risk of breast density – age and post-menopausal body weight, both of which are positively related to risk, are inversely related to density (Boyd *et al*, 1998b; Salminen *et al*, 1998).

Limitations of visual and area-based methods of assessing breast density, such as subjectivity, variations in density with breast compression and X-ray exposure and the time involved in visual assessment of mammograms have led to interest in automated, volumetric methods of breast density. The aim of this study was to explore the use of the Standard Mammogram Form (SMF™) tool (Highnam *et al*, 1996, 1999, 2006; Jeffreys *et al*, 2006; McCormack *et al*, 2007) to investigate relationships between breast cancer risk factors and volumetric breast density.

## MATERIALS AND METHODS

The women included in the study are members of the Glasgow Alumni Cohort (McCarron *et al*, 1999). The cohort was assembled from students at the University of Glasgow (1948–1968) who attended a medical examination at the Student Health Service, at which age at menarche was reported (on average 6 years after the event). Surviving cohort members were contacted by postal questionnaire in 2001, in which women provided information on family history of breast cancer and details of pregnancies, and reported current weight and height, from which we calculated body mass index (BMI). The date or age at last menstrual period (LMP) was asked. Where this was missing, but women reported having had a period in the last 12 months, the age at the time of completing the questionnaire was used as the age at LMP. Self-reported birthweight was asked in pounds and ounces and converted to kilograms for analysis.

Those women living in Scotland were asked to give consent for access to screening mammograms taken under the Scottish Breast Screening Programme (1989–2002). Cranio-caudal and medio-lateral oblique (MLO) films were digitised on site as we have previously described (Jeffreys *et al*, 2006). Both the postal questionnaire survey and the acquisition of digital mammograms received ethical approval from the Multi-centre Research Ethics Committee (Scotland).

For the visual assignment of area density categories, scanned images were displayed at 300-*μ*m resolution on a flat-panel display system. We have previously reported on the similarity of density measures obtained when these assessments are made from the digitised image compared to from the original film (Jeffreys *et al*, 2003). Visual density measures were made by one radiologist experienced in density assessment (RW) using a six-point categorical scale of the percentage of the breast area that appeared dense. The categories were: 0%, 1–10%, 11–24%, 25–49%, 50–74% and ⩾75%; and the scale is referred to in this paper as the six category classification (SCC), a method of visual assessment of breast density. RW has previously reported high agreement with other radiologists in assigning visual density categories to mammograms (Atkinson *et al*, 1999, 2004). The SCC scale was chosen to make our work comparable with that of other researchers, who have found four-fold differences in the risk of breast cancer in women in the extreme categories of this scale (Heine and Malhotra, 2002). The project was initiated prior to the release of the BI-RADS 4 classification system by the American College of Radiology in 2003.

All mammograms for each woman were presented consecutively to the radiologist. Because of differences in density analyses between mammography views (Jeffreys *et al*, 2006; McCormack *et al*, 2007), our analyses were restricted to MLO films. As in previous analyses, we used mammograms taken at the first-screening round a woman attended (Jeffreys *et al*, 2006). To increase the precision of the density assessment, the mean of SMF values or the median SCC category of left and right mammograms taken on this day was used.

### Volumetric density analyses

The volume of dense breast tissue was estimated using the SMF generation programme version 2.2. We have described this in detail previously (Jeffreys *et al*, 2006). In brief, the SMF algorithm models the image formation process to compute at each pixel in the mammogram a measure of the X-ray attenuation and thereby the types and thicknesses of breast tissue in the cone of tissue between the pixel and the X-ray source. The algorithm automatically segments the pectoral muscles to ensure only the breast itself is included in the calculations. This version of SMF assumes that there is only fat and non-fat (‘dense’) tissue in the breast.

Along with the mammogram, SMF requires knowledge of the X-ray imaging parameters in use on the day the mammogram was acquired including exposure current and tube voltage and, ideally, breast thickness and film-processing conditions. If these parameters are not present then the SMF algorithm attempts to estimate them. Errors in these parameters will inevitably cause errors in the SMF values and a sensitivity analysis to investigate such errors has been reported previously (Highnam *et al*, 1996; Highnam and Brady, 1999).

The end result of the SMF algorithm is two volumetric measures of breast density, (i) the absolute volume (cm^3^) of the breast that is dense (SMF volume) and (ii) the percentage of the volume of the breast which is dense (SMF%).

### Statistical analyses

Descriptive analyses report medians (interquartile range (IQR)) for skewed data and means (s.d.) for normally distributed data. Key exposure variables were cross-tabulated against quartiles of SMF volume, SMF% and SCC. Logistic regression models were used to estimate odds ratios (ORs) between exposure variables and breast density, using SMF volume and SMF% dichotomised at the median and SCC split at 50% or greater density compared to under 50% density. Logistic regression was chosen in favour of ordered logistic regression, since initial analyses showed that the common OR for any dichotomy of the outcome variables was not constant, that is, there was not a proportional relationship between the exposure variables and adjacent categories of SMF. All models were adjusted for age (linear variable) at the time of mammography. Confounding was investigated by comparing the magnitude of the estimates from age-adjusted and further adjusted models. Interaction models to test for the presence of a differing relationship between each of the risk factors and pre- compared to post-menopausal breast density were performed. For all models, the linear term for the risk factor was used, rather than the categorical variable, with the exception of birthweight, which appeared to show a quadratic relationship with SMF% and therefore it would have been inappropriate to test the linear association.

## RESULTS

There were 3566 women in the original Glasgow Alumni Cohort, of whom 2169 (61%) were sent a postal questionnaire in 2001. These were the women who could be traced through the National Health Service Central Register and were still alive. The response rate was 59% (*n*=1285). Of the respondents, 935 women (73%) were still living in Scotland. Two hundred and seventy-seven of these women (30%) had never had a screening mammogram, and two women refused access to their films.

The SMF algorithm was run on all 3968 mammograms belonging to 649 of the remaining 656 women (films of seven women were omitted inadvertently). The SMF programme failed on one image (<0.1%) and produced an invalid result for 29 (1.4%) further images. These invalid results can arise from a lack of data, for example, an inability for compute breast thickness if this was not recorded in the medical records, or can occur if the breast did not fit onto one film. Twenty-three (3.5%) women (122 images) were excluded as they reported having had breast cancer in the 2001 questionnaire. Thirty-one women (134 mammograms) had attended university after 1968 so were excluded, since the proportion of students attending the Student Health Service fell dramatically after this date (McCarron *et al*, 1999). Six women (65 images) were excluded because the digitised image was too pale for visual density categories to be assigned. Analyses are based on the MLO images taken at the first-screening visit (*n*=1199) of the remaining 590 women.

When the women attended the University of Glasgow Student Health Service, their median age was 18.7 years (range: 16.8–33.3). The median age at the time of responding to the questionnaire was 61.7 years (range: 51.0–77.8). The median age at first breast screening was 54.1 years (range: 40.0–71.5), including eight women who were over 65 years at the time of their first mammogram.

The distribution of the volume of dense tissue (SMF) was positively skewed. The median volume of dense tissue was 70.25 cm^3^ (IQR: 51.0–103.0). The percentage of the volume of breast tissue that is dense (SMF%) approached a normal curve, with a mean of 26.3% and s.d.=8.0% (range: 12.7–58.8%). Fifty-one women (8.6%) fell into the lowest SCC category (0%) and 110 (18.6%) in the highest category (over 75%). Using the dichotomised SCC variable, 38.9% were classified as having dense breasts (over 50% dense). Relationships between the SMF measures and SCC have been described previously (Jeffreys *et al*, 2006); in summary, SMF%, but not SMF volume, was positively related to SCC. Across SCC categories, the median SMF% rose linearly from 16% (IQR: 14.1–18.1%) to 34.4% (IQR: 29.1–40.4%).

Reproductive and anthropometric characteristics of the included women are shown in Table 1. Over half of the women had their first period at age 12 or 13. One-third of women had their LMP before the age of 50, and a further 47% had their LMP between the ages of 51–55 years. Excluding the 32 women with missing data on LMP, 417 (75%) reported that their LMP was before their first-screening mammogram. These 32 women were considered post-menopausal at the time of their first mammogram for subsequent analyses.

Two-thirds of the women included had ever been pregnant, with the majority of these women having had two or three pregnancies. Most women had their first pregnancy between the age of 24 and 30 years, reflecting the delay in childbearing among university graduates. Forty-seven women reported their mother having had breast cancer; the 50 women who did not answer this question were assumed to have a negative family history of breast cancer in subsequent analyses. Over half of the women did not report their birthweight. Of those who did, the majority, were between 3.0 and 3.9 kg. Under 10% of the women were overweight (BMI ⩾25 kg m^−2^) when at university, by the time the women were aged 51–78 years, this proportion approached 40%.

The age-adjusted associations between breast cancer risk factors and the three measures of high-risk breast density are shown in Table 2. There was no relationship between age at menarche and any of the measures of breast density. Age at LMP was positively related to the percentage of dense breast area and, to a lesser extent, to the percentage of dense breast volume, but was not related to the total volume of dense tissue.

For pregnancy-related variables, SMF volume was most strongly related to the exposures ever having been pregnant and the number of pregnancies, in the same direction as is evident between the variables and breast cancer. Neither SCC nor SMF% was related to these exposures. In contrast, SCC was the only one of the three variables that was related to age at first pregnancy.

There was a suggestion that women whose mothers had had breast cancer had a higher risk of high SMF volume, but not SMF% or SCC. Birthweight appeared to have a quadratic relationship with SMF%, with higher risks of high density seen in women born between 2.5 and 3.9 kg, and significantly lower risks apparent in women born under 2.5 kg or over 4 kg. These results persisted following adjustment for current weight. However, testing the significance of a quadratic term for birthweight gave nonsignificant results, *P*=0.21.

The relationship between BMI and breast density differed according to whether the total volume or percentage volume/area measure was used. Women with a high BMI had a higher risk of absolute SMF volume but a lower risk of SCC and SMF%, the latter because of the high proportion of fat in the breasts of women with a high BMI. These relationships were not affected by adjustment for reproductive risk factors. The magnitude of these relationships was similar for BMI measured in early and later adulthood.

Consideration of the differential effects of breast cancer risk factors on volumetric breast density according to menopausal status is shown in Table 3. These analyses are based on 141 pre- and 449 post-menopausal women. The relatively small numbers may account for the lack of formal tests of interaction not reaching statistical significance, despite clear differences in the magnitude and direction of some of the ORs.

In general, the patterns of association described above were only present for post-menopausal women. For example, post-menopausal women whose menarche had been early had a higher risk of high SMF%, whereas this was not seen for pre-menopausal women, *P* (interaction)=0.079. Similarly, ever having been pregnant and the number of pregnancies was inversely associated with SMF volume in post-menopausal but not pre-menopausal women. Comparing the magnitude of the ORs, there was a suggestion that a maternal history of breast cancer was associated with a higher risk of high SMF volume in pre- but not post-menopausal women, but this was based on only 14 pre- and 31 post-menopausal women with a maternal history of breast cancer. Similarly, the presence of an inverse relationship between birthweight and SMF% was only seen in post-menopausal women. Finally, the positive associations of BMI with SMF volume and the inverse associations with SMF% were seen in all women.

## DISCUSSION

The results presented in this paper highlight for the first time differences in relationships between breast cancer risk factors and several measures of breast density, both area-based and volumetric. The most consistent of these was SMF volume, which was positively associated with higher BMI, ever being pregnant, having had more children and maternal breast cancer. Higher SCC and SMF% were associated with later age at LMP and lower BMI, which themselves lower the risk.

Two limitations of the study are important. First, the SMF method, using GenerateSMF version 2.2, groups all non-fatty tissue, including fibrous (both intra- and extra-lobular), glandular and vascular tissues together, which we refer to as ‘dense’, whereas ideally, the estimated dense volume should only be of glandular tissue. Thus, the computed volumes include both the epithelial tissue components considered relevant to risk, as well as non-epithelial dense components, (including collagen density and stromal composition), less clearly related to risk (Alowami *et al*, 2003, Li *et al*, 2005). If these non-epithelial dense components are constant across levels of exposures (risk factors) studied, their presence should not affect our ORs, although they will distort the estimated volume of true glandular tissue. Additional non-glandular components of dense tissue may vary across levels of risk factors with consequent biased results. More sophisticated modelling to identify these components is in progress.

A second limitation of the study is that, despite theoretical predictions, we do not know the predictive value of SMF. We have previously reported that SMF% correlates well with a frequently used visual assessment of density (Jeffreys *et al*, 2006). Visual- and computer-assisted methods used to assess breast density have been shown to be more strongly related to breast cancer than any other risk factor (Boyd *et al*, 1998a). Investigation of the magnitude of the association between SMF and breast cancer risk is ongoing in a large case–control study. If the mechanism through which high breast density relates to breast cancer risk is due to the amount of glandular breast tissue, we would expect that the volume of breast tissue, if accurate, would be more closely related to risk than would SCC. An SMF measurement system which also removed non-glandular tissue from the volume estimations would probably be even more powerful.

The results which we found for SMF volume and SMF% are broadly consistent with those reported by others in relation to the percentage of the area of the breast which is dense. Previous studies have found positive relationships between age at menarche and breast density (El-Bastawissi *et al*, 2000; Sala *et al*, 2000), although in this and other previous studies (Jakes *et al*, 2000; Maskarinec *et al*, 2002; Heng *et al*, 2004) such associations have not been demonstrated. Lower parity and later age at first pregnancy are two of the most consistently reported risk factors for breast cancer (Kelsey *et al*, 1993), and have also been shown to be related to breast density in later life (Heine and Malhotra, 2002), most recently using both SCC and SMF methods in a sample of 250 women in England (McCormack *et al*, 2007). Our results relating density to parity were mixed, the association with age at first pregnancy being evident only for SCC, and was weaker than reported previously (Heine and Malhotra, 2002). This may reflect the narrower range of age at first pregnancy in this cohort of women who attended university, and delayed having children until a later age.

We found a relationship between SMF volume, but not SMF%, and a maternal history of breast cancer, although this did not quite reach conventional levels of statistical significance. This accords with a recent review, which noted that most relationships reported are weak, and that family history and mammographic density are independent risk factors (Heine and Malhotra, 2002). We had no information on breast cancer in other relatives. Women with a family history of breast cancer may have higher levels of post-menopausal density due to a failure to decrease density over the menopausal period (Knight *et al*, 1999). Although plausible, it is not supported by our observation that the association between maternal history of breast cancer and SMF volume was stronger in pre- than in post-menopausal women. Longitudinal data are required. As screening mammograms are offered in the United States to women aged 40 years and over, and to women in New Zealand from age 45 years, these countries, or the United Kingdom Age Trial of mammography at ages 40–50 years may allow peri-menopausal investigation of breast density.

Our most interesting result concerns current BMI, a well-established post-menopausal risk factor (Lahmann *et al*, 2003). All previous studies have found BMI inversely related to percent breast density (Heine and Malhotra, 2002). Using descriptive parenchymal patterns (e.g. Wolfe) or the percentage of the mammogram (area or volume) which is dense, such observations are inevitable: inherent in the definition of percentage density, fatty areas or volumes are considered not dense; and therefore, present more in women with a high BMI. Our estimation of dense tissue volume, independently of the fat volume, is a significant advantage in understanding whether density is an intermediate step in the relationship between BMI and risk.

Despite continuing reported associations between risk factors and breast density, such work needs to be refined. First, the relative amounts of dense and non-dense tissues should be considered as two separate outcomes, as suggested previously (Boyd *et al*, 1998b) although associations between risk factors and absolute values of density (either volume or area) are often omitted, with a concentration on percent density. Inclusion of both outcomes improves understanding of the determinants of breast density, and their influence on the results.

Second, the mechanisms underlying associations between breast density and risk need investigation. Differences in an area-based measure of breast density and level of acculturation by Chinese women in the United States has been found (Tseng *et al*, 2006). This was only partially explained by risk factors (primarily parity and dairy food consumption). The biological basis for the relationship between breast density and breast cancer is not well understood. One possibility is that insulin-like growth factor (IGF) and its main binding protein IGF-binding protein-3, themselves and their genetic determinants both of which have been related to breast density (Guo *et al*, 2001; Maskarinec *et al*, 2003; Tamimi *et al*, 2007), may play a role. Longitudinal studies of changes in breast density might help here (Salminen *et al*, 1999). Our findings relating to modification by menopausal status were limited by relatively small numbers. Stronger relationships with density in post-menopausal women suggest that some risk factors have a long-term effect, as in a Minnesota study, (Cerhan *et al*, 2005).

In summary, our findings suggest that the novel technique of estimating the volume of dense breast tissue, which involves computerised modelling of mammographic breast density using a fully automated system, may be useful in large epidemiological studies. Work is underway on whether SMF can predict breast cancer risk.

## Figures and Tables

**Table 1:** Characteristics of 590 women from the Glasgow Alumni Study who attended breast screening in Scotland

	***n***	**%**
*Age at menarche*		
10–11 years	63	10.7
12–13 years	346	58.6
14–18 years	180	30.5
Missing	1	0.2
		
*Age at LMP*		
<45 years	67	11.4
45–49 years	136	23.1
50–54 years	277	47.0
⩾55 years	78	13.2
Missing	32	5.4
		
*Ever pregnant*		
Yes	404	68.5
No	183	31.0
Missing	3	0.5
		
*No. of pregnancies*		
0	183	31.0
1	46	7.8
2	132	22.4
3	129	21.9
4+	97	16.4
Missing	3	0.5
		
*Age at first pregnancy*		
⩽23	42	7.1
24–26	136	23.1
27–30	150	25.4
31–35	51	8.6
⩾36	25	4.2
Never pregnant	183	31.0
Missing	3	0.5
		
*Maternal breast cancer*		
Yes	47	8.0
No	493	83.6
Missing	50	8.5
		
*Birthweight (kg)*		
<2.5	16	2.7
2.5–2.9	31	5.3
3.0–3.9	197	33.4
⩾4.0	29	4.9
Missing	317	53.7
		
*BMI in 2001 (kg m*^*−2*^)		
⩽22	121	20.5
22.1–25	216	36.6
25.1–28	121	20.5
>28	107	18.1
Missing	25	4.2
		
*BMI at university (kg m*^*−2*^)		
⩽22	368	62.4
22.1–25	156	26.4
25.1–28	47	7.8
>28	9	1.5
Missing	10	1.7

**Table 2:** Relationship between breast cancer risk factors and high-risk breast density among 590 women in the Glasgow Alumni Cohort

	**SCC**		**SMF volume**		**SMF%**	
	**OR**	**95% CI**	**OR**	**95% CI**	**OR**	**95% CI**
*Age at menarche*						
10–11 years	ref		ref		ref	
12–13 years	0.97	0.55–1.70	0.62	0.36–1.07	0.96	0.56–1.66
14–18 years	1.25	0.68–2.29	0.69	0.38–1.24	0.95	0.53–1.71
Per year	1.06	0.92–1.23	0.94	0.82–1.08	0.97	0.84–1.12
*P* (trend)		0.41		0.39		0.67
						
*Age at LMP*						
<45 years	ref		ref		ref	
45–49 years	1.84	0.92–3.71	1.33	0.74–2.39	1.02	0.56–1.85
50–54 years	2.82	1.48–5.34	1.13	0.66–1.92	1.61	0.93–2.78
⩾55 years	4.20	1.98–8.93	0.99	0.51–1.90	2.33	1.19–4.57
Per year	1.07	1.03–1.11	1.02	0.97–1.03	1.05	1.01–1.08
*P* (trend)		<0.001		0.92		0.004
						
*Ever pregnant*						
Yes	0.89	0.61–1.31	0.48	0.33–0.69	1.03	0.72–1.48
No	ref		ref		ref	
						
*No. of pregnancies*						
0	1.10	0.55–2.18	1.04	0.53–2.04	1.21	0.62–2.35
1	ref		ref		ref	
2	1.15	0.58–2.32	0.53	0.27–1.05	1.43	0.72–2.81
3	0.83	0.41–1.68	0.46	0.23–0.91	1.05	0.53–2.07
4+	0.95	0.45–2.00	0.38	0.19–0.79	1.42	0.69–2.89
Per year	0.95	0.86–1.06	0.78	0.70–0.86	1.01	0.91–1.12
*P* (trend)		0.37		<0.001		0.84
						
*Age at first pregnancy*						
⩽23	0.54	0.25–1.16	1.04	0.52–2.09	0.87	0.43–1.77
24–26	ref		ref		ref	
27–30	0.80	0.49–1.30	0.63	0.39–1.01	0.76	0.48–1.22
31–35	1.27	0.65–2.47	0.67	0.35–1.29	1.26	0.65–2.45
⩾36	1.44	0.60–3.52	1.29	0.55–3.06	0.49	0.20–1.20
Per year	1.06	1.01–1.11	0.99	0.95–1.04	0.99	0.95–1.04
*P* (trend)		0.029		0.82		0.75
						
*Maternal breast cancer*						
Yes	1.38	0.74–2.56	1.70	0.92–3.14	1.36	0.74–2.50
No	ref		ref			
						
*Birthweight (kg)*						
<2.5	0.31	0.09–1.06	1.80	0.62–5.21	0.27	0.08–0.87
2.5–2.9	1.17	0.53–2.55	2.58	1.13–5.93	1.32	0.61–2.88
3.0–3.9	ref		ref		ref	
⩾4.0	0.47	0.19–1.16	1.37	0.62–3.05	0.40	0.17–0.92
						
*BMI in 2001 (kg m*^*−2*^)						
⩽22	1.48	0.92–2.36	0.73	0.46–1.16	1.94	1.20–3.12
22.1–25	ref		ref		ref	
25.1–28	0.39	0.24–0.64	2.50	1.58–3.96	0.80	0.51–1.25
>28	0.25	0.14–0.45	3.65	2.22–6.02	0.24	0.14–0.41
Per kg m^−2^	0.83	0.79–0.88	1.21	1.15–1.28	0.83	0.79–0.88
*P* (trend)		<0.001		<0.001		<0.001
						
*BMI at university (kg m*^*−2*^)						
⩽22	ref		ref		ref	
22.1–25	0.43	0.28–0.65	1.85	1.26–2.70	0.54	0.37–0.79
25.1–28	0.36	0.17–0.74	2.45	1.30–4.65	0.37	0.19–0.71
>28	a		2.62	0.65–10.67	a	
Per kg m^−2^	0.79	0.73–0.85	1.17	1.09–1.26	0.82	0.76–0.88
*P* (trend)		<0.001		<0.001		<0.001

Ref=reference category.

High-risk breast density is defined as over 50% dense for SCC and the upper 50% of the distribution of SMF and SMF%. See text for further details.

All OR are adjusted for the age of the women when the mammogram was taken.

a None of these women had high density mammograms.

**Table 3:** Interaction with menopausal status: relationship between breast cancer risk factors and high-risk volumetric breast density among 590 women in the Glasgow Alumni Cohort

	**SMF volume**				**SMF %**			
	**Pre-menopausal**		**Post-menopausal**		**Pre-menopausal**		**Post-menopausal**	
	**OR**	**95% CI, *P* (trend)**	**OR**	**95% CI, *P* (trend)**	**OR**	**95% CI, *P* (trend)**	**OR**	**95% CI, *P* (trend)**
*Age at menarche*								
Per year later	1.08	0.81–1.43, *P*=0.60	0.90	0.77–1.06, *P*=0.20	0.93	0.69–1.25, *P*=0.63	0.98	0.84–1.15, *P*=0.82
	*P* (interaction)=0.27				*P* (interaction)=0.69			
								
*Age at LMP*								
Per year later	0.97	0.84–1.12, *P*=0.68	1.01	0.96–1.05, *P*=0.79	0.97	0.84–1.12, *P*=0.69	1.04	1.00–1.09, *P*=0.054
	*P* (interaction)=0.82				*P* (interaction)=0.079			
								
*Ever pregnant*								
Yes *vs* no	0.60	0.25–1.14, *P*=0.24	0.45	0.30–0.68, *P*<0.001	0.67	0.26–1.75, *P*=0.41	1.08	0.73–1.61, *P*=0.70
	*P* (interaction)=0.56				*P* (interaction)=0.34			
								
*No. of pregnancies*								
Per pregnancy	0.93	0.74–1.18, *P*=0.56	0.74	0.66–0.83, *P*<0.001	0.92	0.72–1.18, *P*=0.50	1.02	0.92–1.14, *P*=0.67
	*P* (interaction)=0.076				*P* (interaction)=0.35			
								
*Age at first pregnancy*								
Per year later	1.01	0.92–1.11, *P*=0.80	0.99	0.94–1.05, *P*=0.72	1.04	0.94–1.15, *P*=0.42	0.98	0.92–1.04, *P*=0.44
	*P* (interaction)=0.72				*P* (interaction)=0.25			
								
*Maternal breast cancer*								
Yes *vs* no	2.70	0.89–8.24, *P*=0.070	1.37	0.66–2.86, *P*=0.40	1.73	0.52–5.74, *P*=0.36	1.16	0.56–2.41, *P*=0.69
	*P* (interaction)=0.31				*P* (interaction)=0.59			
								
*Birthweight (kg)*								
<2.5	3.17	0.31–32.61	1.53	0.45–5.16	0.49	0.06–3.76	0.20	0.04–0.97
2.5–2.9	4.51	0.88–23.07	1.94	0.73–5.18	0.93	0.24–3.68	1.55	0.60–4.03
3.0–3.9	ref		ref		ref		ref	
⩾4.0	0.29	0.04–2.21	2.09	0.80–5.46	0.20	0.03–1.41	0.49	0.19–1.28
	*P* (interaction)=0.19				*P* (interaction)=0.62			
								
*BMI at university (kg m*^*−2*^)								
Per 1 kg m^−2^	1.26	1.12–1.42, *P*<0.001	1.20	1.13–1.27, *P*<0.001	0.85	0.77–0.94, *P*=0.001	0.83	0.78–0.88, *P*<0.001
	*P* (interaction)=0.49				*P* (interaction)=0.66			
								
*BMI in 2001 (kg m*^*−2*^)								
Per 1 kg m^−2^	1.13	0.97–1.32, *P*=0.10	1.18	1.09–1.28, *P*<0.001	0.75	0.63–0.90, *P*<0.001	0.83	0.77–0.90, *P*<0.001
	*P* (interaction)=0.66				*P* (interaction)=0.33			

High-risk volumetric breast density is based on the upper 50% of the distribution of SMF and SMF%. See text for further details.

All OR are adjusted for the age of the women when the mammogram was taken.
